# Safety and pharmacokinetics of novel selective vascular endothelial growth factor receptor-2 inhibitor YN968D1 in patients with advanced malignancies

**DOI:** 10.1186/1471-2407-10-529

**Published:** 2010-10-05

**Authors:** Jin Li, Xinmin Zhao, Lei Chen, Haiyi Guo, Fangfang Lv, Ka Jia, Ke Yv, Fengqing Wang, Chuan Li, Jun Qian, Chunlei Zheng, Yunxia Zuo

**Affiliations:** 1 Department of Medical Oncology, Fudan University Shanghai Cancer Center; Department of Oncology, Shanghai Medical College, Fudan University, Shanghai 200032, PR China; 2 Shanghai Institute of Materia Medica, Chinese Academy of Sciences, Zhangjiang Hi-Tech Park, Shanghai 201203, PR China

## Abstract

**Background:**

YN968D1 (Apatinib) selectively inhibits phosphorylation of VEGFR-2 and tumor angiogenesis in mice model. The study was conducted to determine the maximum tolerated dose (MTD), safety profile, pharmacokinetic variables, and antitumor activity in advanced solid malignancies.

**Methods:**

This dose-escalation study was conducted according to the Chinese State Food and Drug Administration (SFDA) recommendations in patients with advanced solid tumors to determine the MTD for orally administered apatinib. Doses of continuously administered apatinib were escalated from 250 mg. Treatment continued after dose-escalation phase until withdrawal of consent, intolerable toxicities, disease progression or death.

**Results:**

Forty-six patients were enrolled. Hypertension and hand-foot syndrome were the two dose-limiting toxicities noted at dose level of 1000 mg. MTD was determined to be 850 mg once daily. Pharmacokinetic analysis showed early absorption with a half-life of 9 hours. The mean half-life was constant over all dose groups. Steady-state conditions analysis suggested no accumulation during 56 days of once-daily administration. The most frequently observed drug-related adverse events were hypertension (69.5%, 29 grade 1-2 and 3 grade 3-4), proteinuria (47.8%, 16 grade 1-2 and 6 grade 3-4), and hand-foot syndrome (45.6%, 15 grade 1-2 and 6 grade 3-4). Among the thirty-seven evaluable patients, PR was noted in seven patients (18.9%), SD 24 (64.9%), with a disease control rate of 83.8% at 8 weeks.

**Conclusions:**

The recommended dose of 750 mg once daily was well tolerated. Encouraging antitumor activity across a broad range of malignancies warrants further evaluation in selected populations.

**Trial registration:**

ClinicalTrials.gov unique identifier: NCT00633490

## Background

Vascular endothelial growth factor receptors (VEGFRs) are tyrosine kinases, functioning as a central regulator of multiple signaling pathways that control angiogenesis. The VEGFR-family proteins consist of VEGFR-1/Flt-1, VEGFR-2/KDR/Flk-1, and VEGFR-3/Flt-4 [1,2]. VEGFR-2 is thought to be principally responsible for angiogenesis in malignancies [3]. Various VEGFR-2 inhibitors, including receptor-specific antibodies and low molecular weight chemicals such as sorafenib, vandetanib, cediranib, and sunitinib, have recently been developed [4-6]. In addition to the VEGF-A neutralizing antibody, which is already a standard treatment for late-stage colorectal cancer in the USA [7], sorafenib was recently approved by the US Food and Drug Administration for the treatment of renal and hepatic cancers, and sunitinib was approved for the treatment of gastrointestinal stromal tumor (GIST) and renal cell carcinoma. The application of the VEGFR tyrosine kinase inhibitors (TKIs) to gastrointestinal adenocarcinoma remains a challenge, although they have been found to be active for lung, breast, renal, and hepatic cancers, and GIST.

YN968D1 (apatinib) is an orally administered small-molecule receptor TKI with potential antiangiogenic and antineoplastic activities, developed by Advenchen Laboratories, LLC (Northridge, CA, USA). It is a compound derived from PTK787/ZK222584 (Valatinib) and has been shown to demonstrate superior in vivo efficacy compared to valatinib in xenograft study [8]. Apatinib selectively binds to and inhibits VEGFR-2, which may inhibit VEGF-stimulated endothelial cell migration and proliferation and decrease tumor microvascular density (MVD).

On the basis of encouraging preclinical data, we initiated this first-in-human clinical study in patients with advanced solid tumors. The primary objectives were to determine the maximum-tolerated dose (MTD), dose-limiting toxicities (DLT), pharmacokinetic (PK) profiles, and recommended Phase II dose of apatinib. A secondary objective was to document any antitumor activity.

## Methods

### Patient Selection

Patients with histologically or cytologically confirmed advanced solid malignancies for which no standard alternative curative therapy was available were eligible. Other eligibility criteria were age between 18 to 70 years, ability to take medications orally, with or without measurable lesions, no history of other cancers, an Eastern Cooperative Oncology Group (ECOG) performance status between 0 and 2, life expectancy of >3 months. Additionally, patients had to exhibit adequate hematopoietic function (absolute neutrophils count ≥ 1.5 × 10^9^/L, hemoglobin, ≥90 g/L, platelets, ≥100 × 10^9^/L), hepatic function (bilirubin ≤1.5 × upper limit of normal (ULN), alanine aminotransferase ≤ 2.5 ×ULN, aspartate aminotransferase <2.5 × ULN), renal function(serum creatinine ≤ 1.5 ×ULN, creatinine clearance rate ≥ 50 ml/min), and coagulation function (normal prothrombin time, activated partial thromboplastin time, thrombin time, and fibrinogen). Patients were excluded if they were pregnant or nursing, had a known history of brain metastasis, hypertension, coronary disease or other significant cardiovascular disease, gastrointestinal disorder or other factors that could interfere with drug absorption, were on anti-coagulation therapy, had prior therapy with anti-VEGF or anti-VEGFR targeting agents. Patients with a history of any other malignancy, apart from in situ carcinoma of the cervix or basal cell carcinoma of the skin were also excluded. Patients could not have undergone surgery within the last 4 weeks and should not have been treated within the preceding 4 weeks to enrollment with any investigational drug, chemotherapy, radiotherapy, immunotherapy (6 weeks for nitrosureas and mitomycin C). Written informed consent was obtained from all patients. The Fudan University Shanghai Cancer Center Ethic Committee for Clinical Investigation approved the study.

### Study Design

The phase I study was designed in accordance with the Chinese State Food and Drug Administration (SFDA) recommendations (Guidance for Industry, 2005). PK evaluations were to be performed for single-dosing and multiple-dosing of which the dose levels were determined after completion of dose-escalation. Three dose levels (low, intermediate and high dose) for single-dosing and one dose level (the recommended dose for phase II) for multiple-dosing study, between 8 to 12 participants for each dose levels, were required to meet the SFDA guidance. A cross-over design to evaluate the effects of food intake was incorporated into the single-dosing assessment, also meeting the SFDA requirement, but data will be presented separately.

Apatinib was provided by Advenchen Laboratories, LLC (Northridge, CA, USA) as capsule to be administered orally daily. A safe starting dose was determined in accordance with SFDA recommendations (Guidance for Industry 2005) following a previously established maximum-tolerated dose (MTD) in dogs for YN968D1 of above 30 mg/kg. Thus, in compliance with the SFDA's recommendation, the starting dose was determined to be 250 mg/day, corresponding to one-fourth of the MTD in dogs. (ClinicalTrials.gov number: NCT00633490).

### Treatment

Dose-escalation cohorts enrolled three to six patients. Intrapatient dose escalation was not allowed. DLT was defined as grade 4 hematologic adverse event (AE), or grade ≥3 non-hematologic AE (except for nausea and vomiting that could be improved with optimal supportive care, escalation of alkaline phosphatase) in the first 4-week period. If none of the initial 3 patients developed DLT, dose escalation continued. If one of the initial 3 patients developed DLT, three additional patients were enrolled at the same dose level. If none of the 3 additional patients treated at the same dose level developed DLT, dose escalation continued. If 2 or 3 of the initial 3 patients treated at a dose level, or 1, 2, or 3 of the additional 3 patients at a dose level developed DLT, dose escalation ceased. The MTD was defined as the dose having at most two out of six patients experience DLT.

Treatment continued after dose escalation phase until withdrawal of consent, intolerable toxicities, disease progression or death. During the extended treatment period (after the first cycle) dose reduction was allowed if patient experience toxicities.

PK cohorts enrolled 12 to 18 patients for the 3 planned dose level as determined after dose escalation, and the intermediate-dose cohort would continue to enter the multiple-dosing evaluation after wash-out period.

### Evaluation of Safety and Tolerability

Medical records and laboratory tests were obtained during screening. Physical examination, routine laboratory evaluations, and performance status were assessed at baseline and at specified time points throughout the trial. AEs and concomitant medications were recorded at the end of each cycle. The safety evaluation period extended through 30 days from the last dose of study drug or through recovery to grade 1 or better from all acute toxicities associated with drug administration. Toxicity was evaluated and graded according to the NCI-CTC for Adverse Events, version 3.0.

### Evaluation of Antitumor Activity

Patients with measurable disease at baseline were evaluated for tumor response according to the Response Evaluation Criteria for Solid Tumors (RECIST) [9]. Radiographic studies (CT scan or MRI) for disease assessment were conducted at baseline and every 2 cycles (8 weeks) thereafter. The best overall response was reported. Disease control rate was defined as the percentage of patients with complete response (CR), partial response (PR), and stable disease (SD) for at least 8 weeks. The duration of overall response was defined as the period from the initial measurement of complete response (CR) or partial response (PR), whichever occurred first, to the first date of documented disease recurrence or progression. The same method and techniques of assessment were used to characterize each identified and reported lesion at baseline and at subsequent evaluations.

### Pharmacokinetic Analyses

Whole blood samples (1 ml) were collected at 0 (predose), 0.5, 1, 2, 3, 4, 6, 8, 12, 24, 32, and 48 hours for single-dosing evaluation and 0, 0.5, 1, 2, 3, 4, 6, 8, 12, and 24 hours (before the next dose) on days 1, 6, 28, and 56 for multiple-dosing evaluation. After the last dose on day 56, two additional samples were collected at 32 and 48 hours for multiple-dosing assessment. Plasma was collected from the blood samples following centrifugation, and stored at -80°C until analysis.

Apatinib concentrations in plasma were determined using fully validated specific liquid chromatograph/tandem mass spectrometer methods, with a lower limit of quantitation of 1.6 ng/ml. Based on quality-control samples that were assayed along with the samples, the intraday and interday precision for apatinib analytes ranged from 4.6% to 9.0%, and the accuracy ranged from 85.6% to 110%. The PK parameters, including area under the plasma concentration-time curve (AUC), maximum plasma concentration (*C*_max_), time to maximum concentration (*t*_max_), and elimination half-life (*t*_1/2_) were determined by non-compartmental analysis using InnaPhase Kinetica 2000™ (InnaPhase Corp., Philadelphia, PA, USA). The linear-logarithmic trapezoidal method was used to calculate the AUC, and *t*_1/2 λz _was calculated by linear regression of the terminal slope of the logarithmic plasma concentration-time profile.

## Results

### Patients' Characteristics

From August 2007 to March 2009, 46 patients were treated in this trial which was conducted at Fudan University Shanghai Cancer Center. 19 patients were enrolled in the dose-escalation study. Another 27 patients were enrolled in the PK study. The patients' baseline demographic and clinical characteristics are shown in Table 1. The most common tumor locations were gastrointestinal tracts (73.9%). All patients had received at least one prior chemotherapy regimen, 84.7% prior surgery and 27% prior radiation. 45 had measurable lesions at baseline. Patients received a total of 133 cycles of therapy.

**Table 1:** Patient Baseline Demographic and Clinical Characteristics

Characteristic	Patient	
	No	%
Sex		
Male	26	56.5
Female	20	43.5
Age, years		
Median	51	
Range	23-68	
ECOG performance status		
0	8	17.4
1	36	78.3
2	2	4.3
Prior Therapy	46	
Chemotherapy (*including cytokine and TKI*)		
1	21	
2	10	
≥3	15	
Radiotherapy	13	
Surgery	39	
Other (*Endocrine/Intervention*)	7	
Measurability of baseline disease		
Measurable	45	97.8
Unmeasurable	1	2.1
Primary tumor site		
Gastrointestinal tract	34	73.9
Bronchus/Lung	3	6.5
Breast	3	6.5
Other	6	13

Abbreviation: ECOG, Eastern Cooperative Oncology Group

### Dose Escalation

Five dose levels were investigated: 250 mg, 500 mg, 750 mg, 850 mg, and 1000 mg once daily). One additional patient was included in the 250 mg-cohort due to withdrawal of consent. Two DLTs at the dose level of 1000 mg/day were documented. One patient had grade 3 hypertension and the other grade 3 hand-foot syndrome (HFS). Three additional patients were enrolled to the 850 mg-cohorts. None of the total six patients in the 850 mg-cohort experienced DLTs. Therefore, the MTD for this dosing schedule was determined to be 850 mg daily.

### Pharmacokinetics

Plasma samples collected were analyzed by dose cohort (in mg/day). Eleven patients of the 750 mg-single-dose cohort continued to be enrolled to the 750 mg-multiple-dose cohort after a 7-day-washed-out period while one withdrew consent. Thus, the PK analysis population for single-dosing was n = 8 for 500 mg-cohort, n = 12 for 750 mg-cohort, n = 8 for 850 mg-cohort (one patient from the dose-escalation cohort provided consent to participate in the PK cohort), and for multiple-dosing (750 mg-cohort) n = 11 for 56 days.

Twenty eight patients were assessable for PK analysis. Overall, mean PK parameters of apatinib after single and multiple oral dose administration are summarized in Table 2. For single dose evaluation, C*_max _*was achieved in 3 to 4 hours after oral administration. The plasma levels of apatinib varied considerably between patients. For instance, the C*_max _*values varied between 926 and 4625 ng/ml after a single dose of 750 mg. There was high inter-patient variability. The concentrations of apatinib in plasma increased with dose. C*_max _*and AUC_0-24 _showed a dose-dependent increase at doses from 500 to 850 mg, whereas increased slightly more than dose proportionally at dose of 850 mg, and showed high inter-individual variability. The elimination half-life of the terminal phase (*t*_1/2 λz_), estimated to be approximately 9 hours, was constant over the three dose levels.

**Table 2:** Noncompartmental Mean Pharmacokinetic Parameters of Apatinib After Single or Multiple Oral Doses Administration

PK parameter	Single Oral Dose			Multiple Oral Dose of 750 mg			
		
	500 mg (*n *= 8)	750 mg (*n *= 12)	850 mg (*n *= 8)	Day 1 (*n *= 11)	Day 6 (*n *= 11)	Day 28 (*n *= 11)	Day 56 (*n *= 11)
*C_max_*, ng/ml							
Geometric mean	1,521	2,379	2,833	2,421	2,553	2,210	1,854
CV%	75.1	55.9	90.0	68.5	52.8	45.5	51.2
*t_max_*, hour							
Median	3.5	3.0	4.0	3.0	4.0	4.0	3.0
Range	3.0-8.0	2.0-4.0	1.5-8.0	2.0-8.0	3.0-8.0	2.0-6.0	2.0-6.0
AUC_0-24_, ng·h/ml							
Geometric mean	11,295	18,172	21,975	19,399	25,449	19,946	15,629
CV%	69.7	59.3	80.8	60.5	59.2	43.2	63.2
*t*_1/2λz_, hours							
Mean	8.1	9.0	9.1	8.9	11.0	11.3	8.3
CV%	30.7	15.1	33.1	25.8	56.7	66.5	61.0

Abbreviations: *C_max_*, maximum plasma concentration; CV, coefficient of variation; *t_max_*, time to reach *C_max_*; AUC_0-24_, area under plasma concentration-time curve from 0 to 24 hour; *t*_1/2λz_, half-life associated with terminal slope of a semilogarithmic concentration-time curve.

For multiple-dose evaluation at 750 mg, the mean *C_max _*was 2421 ng/ml on day 1, which increased to 2553 ng/ml by day 6, and the mean AUC_0-24 _was 19399 ng·h/ml on day 1, which increased to 25449 ng·h/ml by day 6. There was no further increase in *C_max _*and AUC beyond 6 days of multiple dosing, and this was similar for the mean elimination *t*_1/2_. Steady-state conditions were achieved by day 6, suggesting no accumulation during 56 days of once-daily dosing.

### Toxicity and Tolerability

The safety population comprised all patients who had received at least one dose of apatinib (N = 46). The most frequently observed drug-related AEs were hypertension (69.5%, 29 grade 1-2 and 3 grade 3-4), proteinuria (47.8%, 16 grade 1-2 and 6 grade 3-4), and hand-foot syndrome (HFS) (45.6%, 15 grade 1-2 and 6 grade 3-4). Hypertension was manageable with antihypertensive agents. About 10% of the patients who developed HFS progressed to grade 3. The application of lotions or moisturizers was useful for symptoms relief. Antibiotic creams were introduced when infection was found. The incidence of all treatment-related AE for each dose levels was listed in Table 3. Treatment-related AEs were generally mild or moderate in severity and were manageable.

**Table 3:** Incidence of Treatment-related Adverse Events in the Study Population for Each Cohort

Adverse Event	NCI CTC Severity Grade	Dose Cohort										Total	
		**250 mg**		**500 mg**		**750 mg**		**850 mg**		**1000 mg**			
		No.	%	No.	%	No.	%	No.	%	No.	%	No.	%

Hypertension	1-2	2	50	7	63.6	9	60	9	69.2	2	66.7	29	63
	3-4	-	-			2	13.3	-	-	1	33.3	3	6.5
Proteinuria	1-2	-	-	4	36.4	7	46.67	5	38.5	-		16	34.8
	3-4	1	25.0	1	9	3	20	1	7.7	-	-	6	13
Hand-Food Syndrome	1-2	-	-	4	36.4	7	46.7	4	30.8	-	-	15	32.6
	3-4	-	-	1	9	2	13.3	2	15.4	1	33.3	6	13
Pain	1-2	-	-	4	36.4	4	26.7	3	23.1	1	33.3	12	26.1
	3-4	-	-	2	18.2	2	13.3	-	-	-	-	4	8.7
Thrombocytopenia	1-2	1	25.0	2	18.2	6	40	2	15.4	1	33.3	12	26.1
	3-4	-	-	0	0	-	-	1	7.7	-	-	1	2.2
Fatigue	1-2	-	-	1	9	4	26.7	4	30.8	1	33.3	10	21.7
	3-4	-	-	-	-	1	6.7	-	-	1	33.3	2	4.3
Hyperbilirubinemia	1-2	-	-	-	-	-	-	3	23.1	-	-	3	6.5
	3-4	1	25	-	-	-	-	-	-	-	-	1	2.2
Transaminase Increased	1-2	2	50.0	1	9	6	40	3	23.1	1	33.3	13	28.3
	3-4	-	-	1	9	-	-	2	15.4	1	33.3	4	8.7
Hemorrhage	1-2	-	-	4	36.4	5	33.3	1	7.7	-	-	10	21.7
	3-4	-	-	1	9	-	-	-	-	-	-	1	2.2
Neutropenia	1-2	-	-	2	18.2	7	46.7	4	30.8	3	100	16	34.8
	3-4	-	-	2	18.2	-	-	2	15.4	-	-	4	8.7
Diarrhea	1-2	-	-	1	9	3	20	2	15.4	-	-	6	13
	3-4	-	-	-	-	-	-	-	-	-	-	-	-
Mucosal ulcers	1-2	-	-	2	18.2	2	13.3	2	15.4	2	66.7	8	17.4
	3-4	-	-	-	-	2	13.3	-	-	-	-	2	4.3
Infection	1-2	1	25.0	1	9	4	26.7	3	23.1	1	33.3	10	21.7
	3-4	-	-	-	-	-	-	-	-	-	-	-	-
Dyspnea	1-2	-	-	-	-	-	-	1	7.7	-	-	1	2.2
	3-4	-	-	-	-	-	-	-	-	-	-	-	-
Vomiting	1-2	-	-	0	0	4	26.7	-	-	-	-	4	8.7
	3-4	-	-	-	-	-	-	1	7.7	-	-	1	2.2
Hoarseness	1-2	-	-	1	9	2	13.3	1	7.7	-	-	4	8.7
	3-4	-	-	-	-	-	-	-	-	-	-	-	-
Albinism	1-2	-	-	-	-	1	6.7	-	-	-	-	1	2.2
	3-4	-	-	-	-	-	-	-	-	-	-	-	-
Anemia	1-2	1	25.0	1	9	3	20	2	15.4	-	-	7	15.2
	3-4	-	-	-	-	-	-	1	7.7	1	33.3	2	4.3
Rash	1-2	-	-	1	9	1	6.7	1	7.7	-	-	3	6.5
	3-4	-	-	-	-	-	-	-	-	-	-	-	-
Anorexia	1-2	-	-	2	18.2	3	20	1	7.7	1	33.3	7	15.2
	3-4	-	-	-	-	-	-	-	-	-	-	-	-

Abbreviations: AE, adverse event; NCI CTC, National Cancer Institute Common Toxicity Criteria

For the two patients who experienced DLT, treatment discontinuation and then re-initiation at a reduced dose after recovery from the AEs was required. 15 patients received at least one cycle of apatinib at the original dose. Four patients discontinued treatment during cycle 1, one in the 250 mg dose group because of disease progression and three in the 1000-mg dose group because of intolerable toxicity. Eighteen patients had a dose reduction for different toxicities during extended treatment (1 in 1000 mg group, 13 in 850 mg, and 4 in 750 mg). The mean duration of administration of apatinib for the patients who experienced dose reduction was 5.4 months.

### Tumor Response

Among the 45 patients with measurable disease, 4 withdrew consent due to receiving other therapy, one experienced intolerable toxicity, and three were lost to follow-up. Thus, thirty-seven patients were evaluable for best overall response. PR was noted in seven patients (18.9%), SD 24 (64.9%), with a disease control rate of 83.8% at 8 weeks. Response was shown for patients at each dose level in Table 4. Among the 7 patients who achieved PR, one was diagnosed with GIST, one cancer of unknown primary, one renal cell carcinoma, one gastric cancer, and 3 colon cancer.

**Table 4:** Response Evaluation for Each Dose Cohort

Dose Cohort	Response in evaluable patient (n = 37)				Disease control (%)
	CR	PR	SD	PD	CR+PR+SD
250 mg	0	1	1	1	2 (66.7)
500 mg	0	2	4	3	6 (66.7)
750 mg	0	2	9	0	11 (100)
850 mg	0	2	8	1	10 (90.9)
1000 mg	0	0	2	1	2 (66.7)

Total	0	7	24	6	31 (83.8)

Abbreviations: CR, complete response; PR, partial response; SD, stable disease; PD, progressive disease.

**Figure 1:**
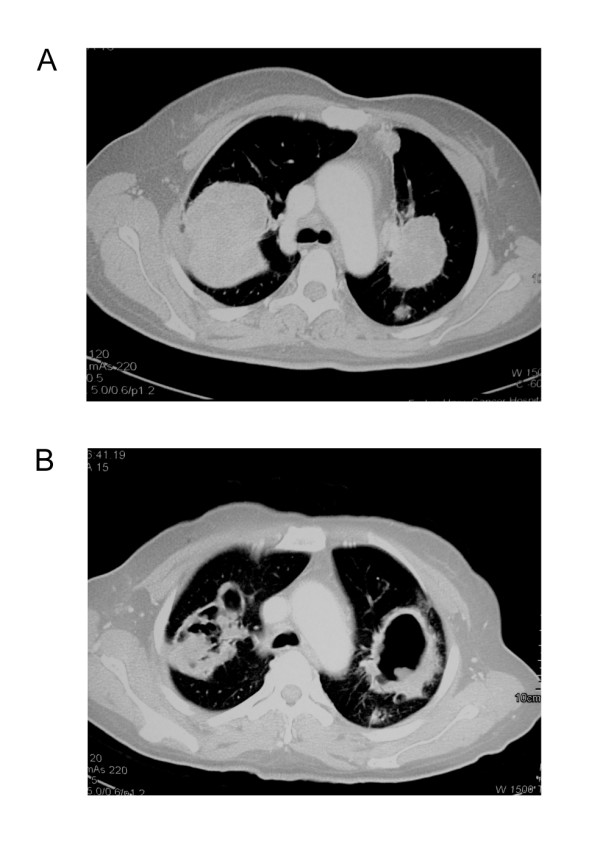
**Computerized tomography scans of the metastatic tumors at baseline (A) and after 2 months (B) showing cavity formation**.

A 45-year-old female with metastatic rectal cancer involving the liver and lung, who had failed prior treatment of 2 cycles of FOLFOX4 regimen, 4 cycles of liver chemoembolization, and 4 cycles of FOLFIRI, treated at 750 mg qd dose level, had a partial response. Compared with the baseline CT-scan, the same lesions on day 53 showed cavity formation and decreased density. Further CT-scan confirmed partial response. Because of hand-foot syndrome, diarrhea and stomatosis, she received reduced dose of 500 mg qd until disease progression (on day 255). Figure 1

**Figure 2:**
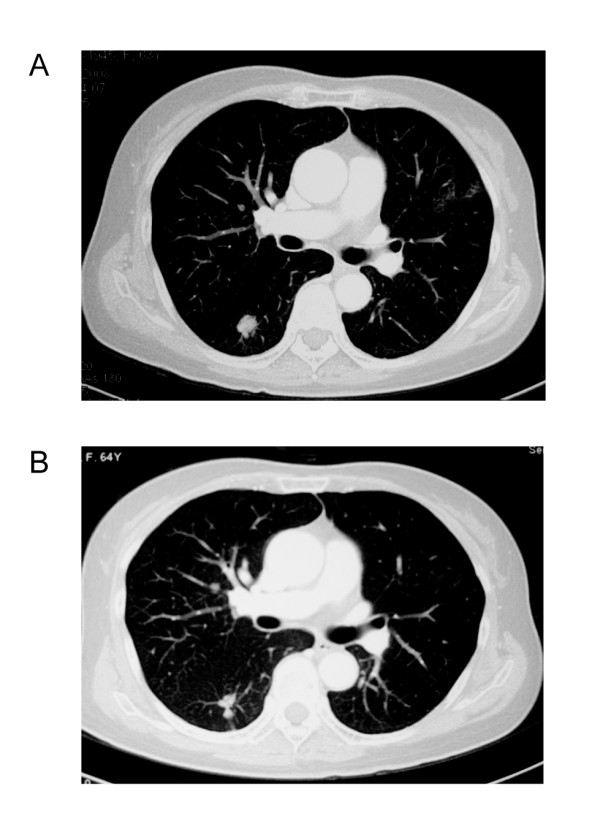
**Tumor shrinkage was confirmed (B) after 4 months of treatment with apatinib comparing with baseline (A)**.

A 65-year-old female with metastatic rectal cancer involving the liver and lung, had prior treatment with 3 cycles of liver chemoembolization and 2 cycles of FOLFOX4 regimen, who refused further chemotherapy, treated at 750 mg qd dose level had a partial response. Compared with the baseline CT-scan, the same lesions on day 60 showed more than 30% decrease in the sum of the longest diameter. Further CT-scan confirmed partial response. The patient received 750 mg qd until disease progression (on day 215). Figure 2

## Discussion

This study demonstrated that hypertension and HFS were the main DLTs. Grade 3 toxicities in first 4 week period were limited to patients receiving apatinib 1000 mg. The PK profile showed that apatinib once daily is orally bioavailable in patients with solid tumors refractory to standard therapy. Apatinib exhibited quick absorption, with *C_max _*reached in 3 to 4 hours. The mean half-life, estimated to be approximately 9 hours, was constant over all dose groups. Steady-state conditions were achieved within 6 days of dosing, with no accumulation during 56 days of once daily dosing of apatinib. These PK and dose-escalation findings support a dose of 750 mg once daily as the recommended phase II dose. Significant interpatient variability with apatinib warrants dose modification to meet individual needs.

Apatinib was well tolerated by most patients at a daily dose of ≤850 mg, and less toxicity greater than grade 3 was experienced by patients receiving the dose under 850 mg of apatinib. The most common AEs were hypertension, proteinuria, and HFS. Hypertension has been observed with all of the oral VEGF TKIs, as reported previously [10]. Therefore, patients with pre-existing hypertension were excluded from this trial as a safety consideration. Systemic hypertension is believed to occur because inhibition of VEGFR in arterial endothelial cells decreases the release of nitric oxide, which acts on arterial smooth muscle cells to cause vasodilation [11]. Although 68% of patients in this study experienced hypertension, it was easily controlled with medication. VEGF is expressed in podocytes in the glomerulus, and VEGF receptors are present on endothelial, mesangial, and peritubular capillary cells. Proteinuria seems to be related to the inhibition of VEGFR and usually regresses with dose reduction. No patients developed glomerulonephritis secondary to apatinib treatment.

Forty-five percent of patients experienced HFS, most of which were grade 1 and 2. Eleven patients (23.9%) experienced bleeding, most of which were found at tumor sites, which is a little higher compared to other VEGFR TKIs. Grade 3 alimentary tract hemorrhage was noted in only 1 patient, mostly attributed to tumor necrosis and active antiangiogenesis. Usually, cessation of apatinib will eliminate this AE due to its relatively short half-life. Although one-third of patients experienced bone marrow suppression, most of these hematologic events were grade 1 or 2 and were manageable. Grade 3-4 neutropenia, thrombocytopenia, and anemia were only seen in 8.7%, 2.2%, and 4.3% of patients, respectively, and these AEs may have been related to apatinib. VEGF receptors are present on bone marrow progenitor cells, which would explain the occurrence of bone marrow suppression [12].

During the extended course of treatment, 38% of patients on reduced dose due to various toxicities continued to experience clinical benefit (disease control) for as long as 5.4 months, suggesting a lower/tolerable dose may be able to achieve greater disease control than the predefined 8 weeks.

Significant antitumor activity was observed in patients with measurable lesions. The majority (n = 31; 83.7%) of these assessable patients exhibited either tumor shrinkage or stabilization according to the RECIST criteria, which is better than those of other TKIs. For sorafenib, an SD of only 26% was reported in the phase I study [6]. In a pooled analysis of 137 patients from 4 phase I trials of sorafenib, only 2 evaluable patients (1.4%) achieved PR and 38 (28%) had SD. Most of the patients (70.8%) showed signs of disease progression by radiological imaging [13]. Strong inhibitory effect on VEGFR-2(IC_50 _= 2.43 nM) may play a key role in the more prominent anti-cancer activity noted in apatinib compared to other VEGFR TKIs, but differences due to patient selection could not be ignored, thus further prospective comparative study may be required. Apatinib has shown promising result for GIST, as one GIST patient who failed imatinib achieved PR and have not progressed to date. Duration of PR was 24 months.

Sunitinib is approved as first-line treatment for renal cell carcinoma, with a 31% response rate and a prolonged progression-free survival (PFS) of 11 months [14]. However, Saltz et al recently reported less activity was noted for colorectal cancer with sunitinib monotherapy [15]. Among the 84 patients with colorectal cancer, only 1 achieved PR, and 13 stable disease. Sunitinib did not demonstrate a clinically meaningful single-agent objective response rate for patients with colorectal cancer refractory to standard chemotherapy. Also, sunitinib has not been shown to be active against gastric adenocarcinoma. In a report from ASCO-GI in 2009, sunitinib was evaluated for chemo-refractory metastatic gastric cancer [16]. Only 5 of 52 patients showed tumor control. Compared with sorafenib and sunitinib, apatinib shows good anti-cancer effects for gastric and colorectal cancer. Disease control rate of 81% was noted among the 22 assessable patients with gastric and colorectal cancer (n = 18), among whom 4 achieved PR.

## Conclusions

The results of this study showed that apatinib was safe and well tolerated, and exhibited substantial antitumor activity at the dose of 750 mg once daily. Promising antitumor activity of apatinib in patients with a broad range of advanced solid tumors has been shown in this study and apatinib is currently being evaluated further in ongoing phase II/III trials (NCT00970138).

## Competing interests

The following potential conflicts of interest were declared: Jin Li (research funding from Merck Serono and Astra Zeneca). The other authors declared no conflict of interest.

## Authors' contributions

XZ, LC, CZ, and FL carried out the study during clinical observation and follow up, KJ, YZ, and HG collected the clinical data for analysis. CL, KY, FW performed the pharmacokinetics evaluations. JQ coordinated the presentation of the PK data. JL was responsible for the overall conception and design of the project, for interpretation of the data and editing of the manuscript. All authors read and approved the final manuscript.

## Pre-publication history

The pre-publication history for this paper can be accessed here:

http://www.biomedcentral.com/1471-2407/10/529/prepub
